# Lecanemab over a two-year duration: Key insights from a regional specialty medical center

**DOI:** 10.1016/j.tjpad.2026.100489

**Published:** 2026-01-24

**Authors:** Lisa B.E. Shields, Hust Hannah, Gregory E. Cooper, Theresa Kluthe, Rachel N. Hart, Andrew P. Thaliath, Brandon C. Dennis, Stephanie W. Freeman, Jessica F. Cain, Whoy Y. Shang, Kendall M. Wasz, Adam T. Orr, Christopher B. Shields, Shirish S. Barve, Kenneth G. Pugh

**Affiliations:** a Norton Neuroscience Institute, Norton Healthcare, Louisville, KY 40202 USA; b Norton Research Institute, Norton Healthcare, Louisville, KY 40202 USA; c Department of Pediatrics, University of Louisville School of Medicine, Louisville, KY 40202 USA; d Department of Neurological Surgery, University of Louisville School of Medicine, Louisville, KY 40202 USA; e Department of Medicine, University of Louisville School of Medicine, Louisville, KY 40202 USA

**Keywords:** Alzheimer’s disease, Mild cognitive impairment, Lecanemab, Amyloid-related imaging abnormalities

## Abstract

**Background and objectives:**

The anti-amyloid monoclonal antibody lecanemab (Leqembi®) treats patients with mild cognitive impairment (MCI) or mild dementia due to Alzheimer’s disease (AD). We sought to evaluate the incidence of amyloid-related imaging abnormalities) ARIA and other adverse events associated with lecanemab.

**Design, setting, and participants:**

This retrospective and observational study features 187 patients who received at least one lecanemab infusion at our multidisciplinary Norton Neuroscience Institute Memory Center over a two-year duration (August 25, 2023-August 24, 2025).

**Results:**

A total of 109 (58.3 %) patients were diagnosed with MCI, and 78 (41.7 %) had mild dementia prior to starting lecanemab. The mean age at the initial infusion was 73 years (Range: 49-90 years). Most (127 [67.9 %]) patients were female, and the majority (181 [96.8 %]) were Caucasian. Of the 175 patients who underwent at least one surveillance brain MRI following lecanemab initiation, 39 (22.3 %) had evidence of ARIA (both ARIA-H and ARIA-E: 13 [33.3 %]; solitary ARIA-H: 17 [43.6 %]; and solitary ARIA-E: 9 [23.1 %]). Of these 39 patients, 20 (51.3 %) were ε4 heterozygous, 12 (30.8 %) were ε4 homozygous, and 7 (17.9 %) were ε4 non-carriers. Patients who were ε4 homozygous more frequently had evidence of any ARIA (p-value = 0.002), ARIA-E (p = 0.041), and ARIA-H (p = 0.004). Of the 25 patients who underwent at least one surveillance brain MRI and were ε4 homozygous, 12 (48.0 %) had ARIA detected. Five (12.8 %) patients with ARIA were symptomatic, requiring lecanemab suspension. Three of these symptomatic patients were ε4 homozygous, and two were ε4 heterozygous. The ARIA was most frequently detected on the surveillance brain MRI performed before the 5^th^ infusion (29 [74.4 %] patients). All 39 cases of ARIA occurred before the 14^th^ lecanemab infusion. Patients with more baseline microbleeds more frequently developed any ARIA (ARIA-H and ARIA-E) (p = 0.041) and solitary ARIA-H (p = 0.022). The presence of baseline microbleeds was associated with a higher frequency of solitary ARIA-H, though was only marginally statistically significant (p = 0.051). Sixty (32.1 %) patients experienced infusion-related adverse effects, with 54 (90.0 %) occurring after the first lecanemab infusion. Mild and transient headaches were most common, affecting 26 (48.1 %) of these patients after the first infusion. After initiating a pre-infusion oral cocktail of acetaminophen 650 mg, loratadine 10 mg, and famotidine 20 mg, the number of patients who experienced an infusion-related adverse event decreased from 45.2 % to 28.3 %. Thirty-two (17.1 %) patients discontinued lecanemab, primarily due to cognitive decline associated with progressive AD (10 [31.2 %]) and ARIA progression (9 [28.1 %]). Of the 73 patients who had MMSE scores performed at baseline and after 1 year post-lecanemab, 13 (17.8 %) had increased scores, 51 (69.9 %) had decreased scores, and the scores remained the same in 9 (12.3 %) patients.

**Conclusions:**

Our findings suggest that ARIA is a significant concern especially in patients who are ε4 homozygous. Close monitoring of patients who are ε4 carriers is recommended to recognize any complications that may ensue.

## 1 Introduction

In 2025 over 7 million Americans are living with Alzheimer’s disease (AD) which is projected to rise to approximately 13 million by 2050 [1]. Health and long-term care costs for individuals with dementia are estimated to be $384 billion in 2025 and is projected to reach $1 trillion in 2050. The anti-amyloid monoclonal antibody lecanemab (Leqembi®) was approved by the U.S. Food and Drug Administration on July 6, 2023 to treat patients with mild cognitive impairment (MCI) or mild dementia due to AD [2,3]. Lecanemab decreased amyloid markers in mild dementia which led to a moderately slower rate of decline in measures of cognition and activities of daily living compared to placebo at 18 months in the phase 3 Clarity AD trial of lecanemab [4]. However, patients may develop amyloid-related imaging abnormalities (ARIA) with microhemorrhages (ARIA-H) or edema (ARIA-E) which may lead to either temporary suspension or permanent discontinuation of lecanemab. Other studies have confirmed that lecanemab may be well-tolerated, reduces the rate of clinical deterioration, and decreases brain amyloid-beta plaques, though infusion-related side effects and/or ARIA may arise [[5], [6], [7], [8], [9], [10], [11]]. Appropriate use recommendations have been published to address the challenges that providers may face when prescribing this drug and provide guidance regarding the surveillance and management of ARIA [2]. Clinicians, patients, and caregivers should be cognizant of the associated risks of lecanemab and closely monitor patients for adverse events throughout the duration of treatment [12].

Our Memory Center previously reported our initial experience treating patients with lecanemab over a six-month period (August 25, 2023 to March 1, 2024) [13]. Almost 25 % of the patients who completed one or more safety monitoring brain MRIs had ARIA detected. Most of these patients were asymptomatic and were either ε4 homozygous or ε4 heterozygous. Through our early use of lecanemab, we described several lessons learned from an organizational, clinical, and payment and insurance coverage standpoint [13].

Herein, we have expanded our previous lecanemab study and now report our experience with patients treated with lecanemab for MCI and mild dementia due to AD over a two-year period. The occurrence of ARIA on surveillance brain MRIs following lecanemab initiation is discussed. We also present the number of patients who have discontinued lecanemab and their reasons for doing so. Key insights through our ongoing use of lecanemab as well as future directions of lecanemab are described.

## 2 Methods

### 2.1 Study population and Memory Center team

Under an Institutional Review Board-approved protocol and according to the Declaration of Helsinki, we conducted a retrospective review of 187 patients treated with at least one lecanemab infusion at the Norton Neuroscience Institute Memory Center (NNI-MC) over a two-year duration (August 25, 2023-August 24, 2025). The inclusion and exclusion criteria as well as detailed descriptions of pre-lecanemab testing (CSF biomarkers, amyloid PET scans, baseline brain MRIs, ApoE genotyping, and cognitive testing [Mini-Mental State Examination [MMSE] or Montreal Cognitive Assessment [MoCA]) were previously described in our initial lecanemab publication [13]. Amyloid status was determined by one of the three options depending on individual clinical circumstances: CSF testing, an amyloid PET scan, or PrecivityAD2® (C_2_N Diagnostics; St. Louis, MO) blood test.

Patients were treated with intravenous (IV) lecanemab 10 mg/kg per infusion with an IV infusion administered every 2 weeks. After observing numerous side effects following the lecanemab infusions within the first several months of initiating this drug, every patient was pretreated with an oral cocktail of acetaminophen 650 mg, loratadine 10 mg, and famotidine 20 mg starting on December 31, 2023. After completing 18 months of lecanemab therapy, patients were given the option of receiving the infusion on a monthly basis instead of every 2 weeks [14]. Baseline brain MRIs were performed prior to the initial lecanemab administration, and surveillance MRIs were subsequently obtained before the 5^th^, 7^th^, and 14^th^ infusions [4]. If ARIA-H and/or ARIA-E was detected on surveillance MRIs, the patient required monthly MRIs until ARIA-E resolved or ARIA-H stabilized.

The NNI-MC is associated with Norton Healthcare which serves the regional, metropolitan community of Louisville, Kentucky. The Memory Center clinical capacity has increased exponentially over the past 4 years, from 928 new patients evaluated in 2021 to 2,573 in 2025 annualized. As of September 2025, our NNI-MC team consists of 2 neurologists, 2 geriatricians, 2 physician assistants, 1 neuropsychologist, 1 neuropsychometrist, 1 nurse practitioner, 1 full-time nurse navigator, 4 medical assistants, 1 MRI scheduler, 1 pharmacist, and the authorization team. In addition to the NNI-MC team, several other resources are valuable to effectively and safely treat patients, including the task force administration, infusion members, an MRI scheduler just for the surveillance brain MRIs, and the billing and coding team.

Several metrics were collected including the patient’s age, gender, race, Body Mass Index (BMI), highest level of education, medical specialty of the referring physician, comorbidities, payer of lecanemab, MMSE/MoCA scores, ApoE genotype, medications for MCI/AD (donepezil, memantine, galantamine, rivastigmine), ARIA-H and/or ARIA-E on surveillance brain MRIs, and infusion-related side effects. The severity of ARIA-H and ARIA-E was also determined [2,15,16]. Management of ARIA was based on the phase III Clarity AD trial [4] and the lecanemab appropriate use recommendations [2]. At the 2-year mark of infusing lecanemab, we assessed how many patients continued to be administered lecanemab and the reasons that patients discontinued this drug. A comparison of MMSE scores at baseline and between 1-2 years post-lecanemab was also performed. We initially analyzed these metrics in all 187 patients who received at least one lecanemab infusion. We subsequently performed an evaluation of a subset of patients who had undergone at least one surveillance brain MRI following the start of lecanemab, specifically, patients who were treated with at least 4 lecanemab infusions.

### 2.2 Statistical analysis

Patient demographics, comorbidities, and baseline brain MRI findings were compared between those who had no evidence of ARIA on surveillance brain MRI scans and those who had any ARIA, solitary ARIA-H, and solitary ARIA-E using Kruskal-Wallis tests for the continuous variables and Chi-squared tests or Fisher’s exact test for categorical variables as appropriate depending on the number of subjects per category. ARIA rates and patients who were symptomatic with ARIA were compared between patients with the lowest quartile baseline MMSE scores (≤ 23) and those with the highest quartile baseline MMSE scores (≥ 27) by the same tests described above. A multivariate binomial regression was performed, predicting symptomatic ARIA by the diagnosis (MCI versus mild dementia) and MMSE scores. These tests were performed on R version 4.2.3 (2023-03-15 ucrt).

### 2.3 Ethical approval and informed consent

The WCG Institutional Review Board (IRB) determined that this retrospective study was exempt under 45 CFR 46.104(d)(4). The IRB number is 2024-0001, and the IRB approval was dated January 3, 2024. According to federal regulations, the IRB of record determined that this study was exempt Category 4 with a complete waiver of consent and authorization.

## 3 Results

### 3.1 Clinical characteristics

A total of 109 (58.3 %) patients were diagnosed with MCI, and 78 (41.7 %) had mild dementia due to AD prior to starting lecanemab (Table 1). The mean age at the initial infusion was 73 years (Range: 49-90 years). Most (127 [67.9%]) patients were female, and the majority (181 [96.8%]) were Caucasian. Most (141 [75.4%]) patients had pursued higher education (education beyond high school). Most patients (177 [94.6%]) had been treated with medications for MCI/AD, with donezepil as the most common (164 [87.l7%]).

**Table 1:** Characteristics of patients treated with lecanemab at our Memory Center.

Features	Number of patients (n=187)
**Diagnosis**	
MCI	109 (58.3 %)
Mild dementia	78 (41.7 %)
**Age at 1^st^ lecanemab infusion (years)**	Mean: 73 years (Range: 49-90 years)
< 65	20 (10.7 %)
66-74	86 (46.0 %)
75-84	76 (40.6 %)
> 85	5 (2.7 %)
**Gender**	
Female	127 (67.9 %)
Male	60 (32.1 %)
**Race**	
Non-Hispanic White	181 (96.8 %)
African-American	5 (2.7 %)
Asian	1 (0.5 %)
**Body Mass Index at 1^st^ lecanemab infusion**	Mean: 26.0 (Range: 17.6-45.0)
< 19.9	12 (6.4 %)
20.0-24.9	68 (36.4 %)
25.0-29.9	79 (42.2 %)
30.0-34.9	22 (11.8 %)
35.0-39.9	3 (1.6 %)
> 40.0	3 (1.6 %)
**Highest level of education**	
Did not complete high school	5 (2.7 %)
High school graduate	41 (21.9 %)
Some college	24 (12.8 %)
Technical school after high school	5 (2.7 %)
Associate’s degree	11 (5.9 %)
College graduate	49 (26.2 %)
Post-college degree *	47 (25.1 %)
Unknown	5 (2.7 %)
**Medical specialty of referring physician**	
Primary Care Provider	151 (80.7 %)
Self-referral	17 (9.1 %)
Neurologist	15 (8.0 %)
Other	4 (2.1 %)
**Comorbidities**	
Dyslipidemia	141 (75.4 %)
Hypertension	86 (46.0 %)
Cardiovascular disease (other than hypertension)	60 (32.1 %)
Diabetes mellitus	35 (18.7 %)
**Payer of lecanemab**	
Medicare	143 (76.5 %)
Medicare A and B (“traditional” Medicare)	87 (60.8 %)
Medicare Advantage/Replacement	56 (39.2 %)
Patient Assistance Program	31 (16.6 %)
Commercial insurance	13 (6.9 %)
**Medications for mild cognitive impairment/AD**	
Donepezil	164 (87.7 %)
Memantine	103 (55.1 %)
Galantanime	10 (5.3 %)
Rivastigmine	12 (6.4 %)
None	10 (5.3 %)
**Testing for AD**	
CSF	165 (88.2 %)
PET scan (amyloid)	18 (9.6 %)
Blood test to detect status of amyoid pathology	13 (7.0 %)
**APOE ε4 status**	
ε4 Heterozygote	102 (54.5 %)
ε4 Homozygote	27 (14.4 %)
ε4 Non-carrier	58 (31.0 %)
**Findings on baseline brain MRI**	
Subcortical hyperintensities	165 (88.2 %)
Mild	137 (83.0 %)
Moderate	28 (17.0 %)
Microbleeds	20 (11.4 %)
**Baseline MMSE/MoCA Scores**	
MMSE (n=148 [79.1 %])	Mean: 25 (Range: 19-30)
MoCA (n=39 [20.9 %])	Mean: 21 (Range: 14-28)

MCI: Mild cognitive impairment

AD: Alzheimer’s disease

⁎ Post-college degree: Master’s, PhD, MD, JD

### 3.2 Testing prior to lecanemab infusion

Patients who received lecanemab had either positive CSF biomarkers (165 [88.2 %]), a positive amyloid PET scan (18 [9.6 %]), and/or positive plasma based biomarkers for AD (PrecivityAD2®) (13 [7.0 %]) (Table 1). Nine (4.8 %) patients underwent testing for plasma based biomarkers for AD without undergoing CSF or amyloid PET scan testing.

A total of 102 (54.5 %) patients were ε4 heterozygous, 27 (14.4 %) were ε4 homozygous, and 58 (31.0 %) were ε4 non-carriers. The number of patients with the following findings on baseline brain MRI included: four or fewer microbleeds (17 [9.1 %]) and subcortical hyperintensities (165 [88.2 %]), the latter of which were mild in 137 (83.0 %) patients and moderate in 28 (17.0 %) patients. Of the 148 (79.1 %) patients who underwent MMSE testing and 39 (20.9 %) patients who had MoCA testing, the mean baseline scores were 25 and 21, respectively.

### 3.3 ARIA-H and ARIA-E detected on surveillance brain MRI

**Fig. 1:**
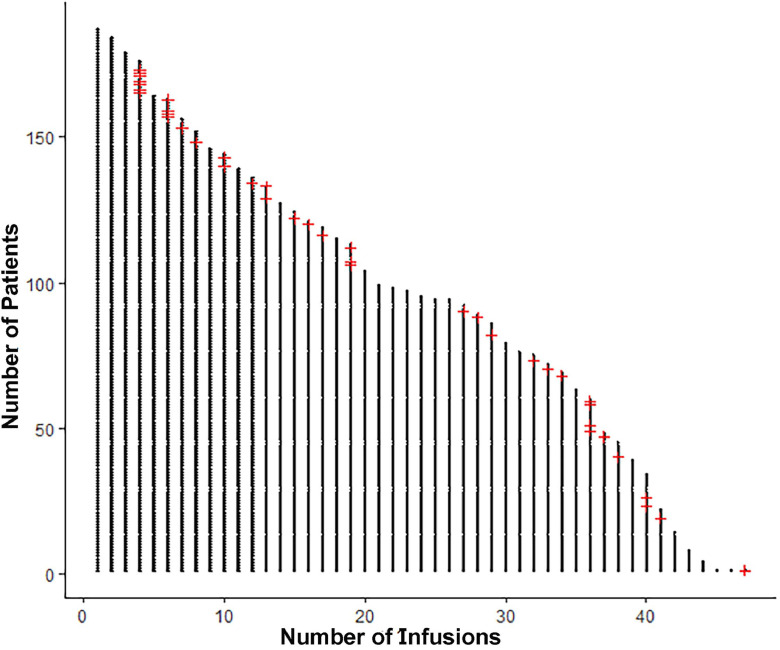
The detection of ARIA-H and ARIA-E on surveillance brain MRIs in relation to the number of lecanemab infusions. Each dot represents one infusion. Each red plus sign indicates the detection of ARIA (n=39).

Fig. 1 depicts when ARIA was detected on surveillance brain MRIs among all patients treated with lecanemab. Of the 175 patients who underwent at least one surveillance brain MRI scan following lecanemab initiation, 39 (22.3 %) had evidence of ARIA (Table 2, Table 3). Both ARIA-H and ARIA-E were present in 13 [33.3 %], solitary ARIA-H was observed in 17 [43.6 %], and solitary ARIA-E was detected in 9 [23.1 %]). Of the 39 patients with ARIA, 20 (51.3 %) were ε4 heterozygous, 12 (30.8 %) were ε4 homozygous, and 7 (17.9 %) were ε4 non-carriers. Patients who were ε4 homozygous more frequently had evidence of any ARIA (ARIA-H and ARIA-E) (p-value = 0.002), solitary ARIA-E (p = 0.041), and solitary ARIA-H (p = 0.004) (Table 4). Of the 25 patients who underwent at least one surveillance brain MRI and were ε4 homozygous, 12 (48.0 %) had ARIA detected. Of the 95 patients who underwent at least one surveillance brain MRI and were ε4 heterozygous, 20 (21.0 %) had ARIA detected. Of the 55 patients who underwent at least one surveillance brain MRI and were ε4 non-carriers, 7 (12.7 %) had ARIA detected. Patients with more baseline microbleeds more frequently developed any ARIA (ARIA-H and ARIA-E) (p = 0.041) and solitary ARIA-H (p = 0.022), as determined by the Kruskal-Wallis Test and operationalized as a continuous variable. The presence of baseline microbleeds was associated with a higher frequency of solitary ARIA-H, though was only marginally statistically significant (p = 0.051).

**Table 2:** Status of patients treated with lecanemab at our Memory Center at the two-year mark.

Features	Number of patients (n=187)
**ARIA-H/ARIA-E detected on surveillance brain MRI (n=175)**	
Yes	39 (20.8 %)
Solitary ARIA-H	17 (43.6 %)
Solitary ARIA-E	9 (23.1 %)
Both ARIA-H and ARIA-E	13 (33.3 %)
**ARIA-H/ARIA-E detected on surveillance brain MRI (n=39)**	
ε4 Homozygote	12 (30.8 %)
ε4 Heterozygote	20 (51.3 %)
ε4 Non-carrier	7 (17.9 %)
**APOE ε4 status and detection of ARIA-H/ARIA-E on surveillance brain MRI**	
ε4 Homozygote (n=25)	12 (48.0 %)
ε4 Heterozygote (n=95)	20 (21.0 %)
ε4 Non-carrier (n=55)	7 (12.7 %)
**ARIA-H/ARIA-E first detected on surveillance brain MRI (n=39)**	
Before 5^th^ infusion	29 (74.4 %)
Before 7^th^ infusion	7 (17.9 %)
Before 14^th^ infusion	3 (7.7 %)
**Infusion-related adverse effects**	
Yes	60 (32.1 %)
No	127 (67.9 %)
**Infusion-related adverse effects following 1^st^ lecanemab infusion and when they started**	
Yes	54 (28.9 %)
At Infusion Center (0–3 h)	14 (25.9 %)
3–24 h post-infusion	36 (66.7 %)
24–48 h post-infusion	2 (3.7 %)
> 48 h post-infusion	2 (3.7 %)
No	133 (71.1 %)
**Infusion-related adverse effects before and after pre-infusion cocktail (December 31, 2023)**	
Before pre-infusion cocktail (n=42)	19 (45.2 %)
After pre-infusion cocktail (n=145)	41 (28.3 %)
**Status of lecanemab infusions**	
Ongoing	155 (82.9 %)
Discontinued	32 (17.1 %)
Progressive cognitive decline/delusions/agitation	10 (31.2 %)
ARIA progression	9 (28.1 %)
Moved to different city/state	3 (9.4 %)
Focus on other medical conditions	3 (9.4 %)
Need for continued anticoagulation	3 (9.4 %)
Surveillance brain MRI detected bilateral SDH	1 (3.1 %)
Pursue conservative treatment (not lecanemab)	1 (3.1 %)
Non-compliant with infusions	1 (3.1 %)
Died after 1^st^ infusion (most likely cardiac-related)	1 (3.1 %)
**Number of lecanemab infusions**	Mean: 24 (Range: 1-47)
1-3	11 (5.9 %)
4-10	37 (19.8 %)
11-20	40 (21.4 %)
21-30	22 (12.3 %)
31-40	56 (29.9 %)
> 40	21 (11.2 %)
**Comparison of MMSE scores at baseline and after 1 year post-lecanemab (n=73)**	
Increased	13 (17.8 %)
Decreased	51 (69.9 %)
Same	9 (12.3 %)

**Table 3:** ARIA-H and ARIA-E in patients treated with lecanemab at our Memory Center.

Patient #	Age/ Gender	Co-morbidities	APOE4 Genotype	Brain MRI Prior to Lecanemab Infusion	Surveillance Brain MRIs	MRIs Following ARIA Detection	Symptomatic/ Lecanemab Permanently Suspended
1	67/M	HTN, HC, DM	ε4/ε4	2 foci cerebral microbleeds	Before 5^th^ infusion: 3 new foci ARIA-H for total of 5 [Moderate]; lecanemab held	1^st^ MRI following ARIA: negative for new ARIA- H and ARIA-E; lecanemab resumed	No
2	80/M	HTN, HC, DM, CVD *	ε3/ε4	2 foci superficial siderosis each less than 1 cm in diameter	Before 5^th^ infusion: 9 new foci ARIA-H [Moderate], unchanged appearance of superficial siderosis (less than 1 cm in diameter); lecanemab held	1^st^ MRI following ARIA: 1 new focus ARIA-H [Severe], unchanged appearance of superficial siderosis (less than 1 cm in diameter); 2^nd^ MRI following ARIA: 1 new focus ARIA-H [Severe]; lecanemab not resumed	No/Yes after the 4^th^ infusion
3	72/M	HC, DM, CVD **; Aspirin 81 mg	ε3/ε4	No evidence of microbleeds or vasogenic edema	Before 7^th^ infusion: 1 focus ARIA-E (8.0 mm in diameter) [Mild]; lecanemab continued	1^st^ MRI following ARIA: negative for new ARIA-H and ARIA-E, previous focus of ARIA-E resolved	No
4	77/F	HTN	ε3/ε3	No evidence of microbleeds or vasogenic edema	Before 7^th^ infusion: 4 new foci ARIA-H [Mild] and 1 new focus ARIA-E (4.0 cm in diameter) [Mild]; lecanemab continued	1^st^ MRI following ARIA negative for new ARIA-H, decreased size of ARIA-E (3.7 cm in diameter), and 1 new infarct (7.0 mm in diameter) in the left cerebellar hemisphere	No
5	74/M	HC	ε3/ε3	No evidence of microbleeds or vasogenic edema	Before 7^th^ infusion: 1 focus ARIA-E (4.2 cm in diameter) [Mild] and 1 focus of ARIA-H [Mild]; lecanemab continued	1^st^ MRI following ARIA: 1 new focus ARIA-E (less than 1 cm in diameter) and increased size of previous focus of ARIA-E (5.8 cm in diameter) [Moderate]; lecanemab suspended; 3^rd^ MRI following ARIA: resolution of ARIA-E and ARIA-H; lecanemab resumed	No
6	71/F	None; Aspirin 81 mg	ε4/ε4	No evidence of microbleeds or vasogenic edema	Before 5^th^ infusion: 6 foci ARIA-H [Moderate] and 1 new focus ARIA-E (6.0 cm in diameter) [Moderate]; lecanemab suspended	1^st^ MRI following ARIA: 5 new ARIA-H [Severe] and decrease in size of previous ARIA-E (3.0 cm) [Mild]	Yes (headaches)/Yes after the 4^th^ infusion
7	71/F	HC	ε3/ε4	No evidence of microbleeds or vasogenic edema	Before 5^th^ infusion: 1 focus ARIA-E (less than 1 cm in diameter) [Mild]; lecanemab continued	1^st^ MRI following ARIA: ARIA-E resolved; 4^th^ MRI following ARIA: 1 focus ARIA-E (less than 5 cm in diameter) [Mild]; 5^th^ MRI after ARIA: increased size of ARIA-E (3.2 cm in diameter) [Mild]; 6^th^ MRI after ARIA: 2 new foci ARIA-E (0.5 cm and 2.2 cm in diameter) [Moderate]; lecanemab suspended after 17^th^ infusion due to headaches and persistent ARIA-E	Yes (headaches)/Yes after the 17^th^ infusion
8	75/F	HTN, HC	ε4/ε4	No evidence of microbleeds or vasogenic edema	Before 5^th^ infusion: 1 focus ARIA-H [Mild]; lecanemab continued	1^st^ MRI following ARIA negative for new ARIA-H and ARIA-E	No
9	74/F	HTN, HC	ε3/ε4	No evidence of microbleeds or vasogenic edema	Before 5^th^ infusion: 1 focus ARIA-E (1.8 cm in diameter) [Mild]; lecanemab continued	1^st^ MRI following ARIA: increased size of ARIA-E (2.2 cm in diameter) and 1 new focus ARIA-E (3.0 cm) [Moderate]; lecanemab suspended	No/Yes after the 36^th^ infusion
10	73/F	HC	ε4/ε4	No evidence of microbleeds or vasogenic edema	Before 5^th^ infusion: 1 focus ARIA-E (3.0 cm in diameter) [Mild] and 4 new ARIA-H [Mild]; lecanemab suspended due to ARIA and symptoms	1^st^ MRI following ARIA: increased size of ARIA-E (3.4 cm) and 4 new foci of ARIA-H [Moderate]; 2^nd^ MRI following ARIA: ARIA-H stable [Mild] and ARIA-E decreased in size [Mild]; 3^rd^ and 4^th^ MRIs following ARIA: resolution of ARIA-H and ARIA-E; lecanemab resumed; 5^th^ MRI following ARIA: 3 new foci ARIA-H [mild]; lecanemab continued; 6^th^ MRI following ARIA: stable ARIA-H [mild]; lecanemab continued	Yes (fatigue/ disorientation for several days after 4^th^ infusion)
11	71/F	HC	ε2/ε4	No evidence of microbleeds or vasogenic edema	Before 5^th^ infusion, 2 new foci ARIA-H [Mild]; lecanemab continued		No
12	74/F	None	ε2/ε4	No evidence of microbleeds or vasogenic edema	Before 5^th^ infusion, 2 new foci ARIA-H [Mild]; lecanemab continued		No
13	63/M	None	ε2/ε3	No evidence of microbleeds or vasogenic edema	Before 14^th^ infusion: 1 new focus ARIA-E (5.0 mm in diameter) [Mild]; lecanemab continued	1^st^ MRI following ARIA: ARIA-E resolved	No
14	64/M	HC	ε4/ε4	No evidence of microbleeds or vasogenic edema	Before 5^th^ infusion: 4 foci ARIA-H [Mild] and 1 new focus ARIA-E (2.0 cm in diameter) [Mild]; lecanemab continued	1^st^ MRI following ARIA: 11 new foci ARIA-H for total of 15 [severe] and increased size of ARIA-E (5.5 cm in diameter) [moderate]; lecanemab discontinued after 6^th^ infusion	No/Yes after the 6^th^ infusion
15	70/F	None	ε3/ε3	No evidence of microbleeds or vasogenic edema	Before 5^th^ infusion: 3 new foci ARIA-H [mild]; lecanemab continued	1^st^ MRI following ARIA: 1 new focus ARIA-H for total of 4 [mild] and 1 new focus ARIA-E (2.0 cm in diameter) [mild]; lecanemab discontinued after 6^th^ infusion	No/Yes after the 6^th^ infusion
16	80/F	HTN, HC	ε3/ε4	1 focus cerebral microbleed	Before 5^th^ infusion: 1 new focus ARIA-H [mild] and 1 focus ARIA-E (3.0 cm in diameter) [mild]; lecanemab continued	1^st^ MRI following ARIA: 2 new foci ARIA-H for total of 3 [mild] and increased size of ARIA-E (4.5 cm in diameter) [mild]	No
17	72/M	None	ε3/ε4	No evidence of microbleeds or vasogenic edema	Before 5^th^ infusion: 1 focus ARIA-H [mild]; lecanemab continued	1^st^ MRI following ARIA: no new ARIA-H [mild]	No
18	77/F	HTN	ε4/ε4	No evidence of microbleeds or vasogenic edema	Before 5^th^ infusion: 1 new focus ARIA-H [mild]; lecanemab continued	1^st^ MRI following ARIA: 1 new focus ARIA-H for total of 2 [mild]; lecanemab held and discontinued after 8^th^ infusion; 4^th^ surveillance MRI: 7 new foci ARIA-H [moderate]	No/Yes after the 8^th^ infusion
19	76/F	HC	ε4/ε4	No evidence of microbleeds or vasogenic edema	Before 5^th^ infusion: 1 focus ARIA-E (sulcal effusion) [mild]; lecanemab continued	1^st^ MRI following ARIA: ARIA-E resolved	No
20	69/F	HC, CV	ε4/ε4	No evidence of microbleeds or vasogenic edema	Before 5^th^ infusion: 1 focus ARIA-H [mild]; lecanemab continued	1^st^ MRI following ARIA: no change in ARIA-H [mild]	No
21	70/M	HTN, HC, CV	ε3/ε3	No evidence of microbleeds or vasogenic edema	Before 5^th^ infusion: 1 focus ARIA-H [mild]; lecanemab continued	1^st^ MRI following ARIA: no change in ARIA-H [mild]	No
22	72/F	None	ε3/ε4	1 focus cerebral microbleed	Before 5^th^ infusion: 2 foci ARIA-E (5.5 cm and 4.2 cm in diameter) [moderate]; lecanemab held	1^st^ MRI following ARIA: decreased size in ARIA-E (3.9 cm and 4.2 cm) [mild]; lecanemab resumed after 4^th^ surveillance MRI; 6^th^ surveillance MRI: 4 new ARIA-E (all with diameter less than 1.0 cm) [moderate]; lecanemab discontinued after 15^th^ infusion	No/Yes after the 15^th^ infusion
23	76/M	HC, CV	ε3/ε4	2 foci cerebral microbleeds	Before 5^th^ infusion: 1 new focus ARIA-H for total of 3 [mild]; lecanemab continued	1^st^ MRI following ARIA: no change in ARIA-H [mild]	No
24	71/F	HTN	ε3/ε4	2 foci cerebral microbleeds	Before 5^th^ infusion: 1 new focus ARIA-H for total of 3 [mild]; lecanemab continued	1^st^ MRI following ARIA: 1 new ARIA-H for total of 4 [mild]	No
25	66/M	None	ε3/ε4	No evidence of microbleeds or vasogenic edema	Before 14^th^ infusion: 3 new foci ARIA-H [mild]; lecanemab continued	1^st^ MRI following ARIA: no change in ARIA-H [mild]	No
26	80/F	HC, DM	ε3/ε4	No evidence of microbleeds or vasogenic edema	Before 5^th^ infusion: 1 new focus ARIA-H [mild] and 1 new focus ARIA-E (2.4 cm in diameter) [mild]; lecanemab held	1^st^ MRI following ARIA: no change in ARIA-H [mild] and decreased size of ARIA-E (1.3 cm in diameter) [mild]; lecanemab discontinued after 4^th^ infusion	No/Yes after the 4^th^ infusion
27	69/M	HC/DM	ε4/ε4	No evidence of microbleeds or vasogenic edema	Before 7^th^ infusion: 5 new foci ARIA-H [moderate] and 1 new focus ARIA-E (1.4 cm in diameter) [mild]; lecanemab held	1^st^ MRI following ARIA: 1 new focus ARIA-H for total of 6 [moderate] and resolution of ARIA-E; lecanemab still suspended; 4^th^ surveillance MRI: no change in ARIA-H [moderate]; lecanemab resumed after 4^th^ surveillance MRI	No
28	86/F	HTN, HC, CV	ε3/ε3	No evidence of microbleeds or vasogenic edema	Before 5^th^ infusion: 3 foci ARIA-H [mild]; lecanemab continued	1^st^ MRI following ARIA: 1 new focus ARIA-H for total of 5 [moderate]; lecanemab held; 3^rd^ surveillance MRI: no change in ARIA-H [moderate] and 1 new focus ARIA-E (0.5 cm in diameter) [mild]; lecanemab still held; 4^th^ surveillance MRI: no change in ARIA-H [moderate] and resolution of ARIA-E; lecanemab resumed after 4^th^ surveillance MRI	No
29	66/M	HTN, HC	ε2/ε4	No evidence of microbleeds or vasogenic edema	Before 7^th^ infusion: 1 new focus ARIA-E (3.7 cm in diameter) [mild]; lecanemab held since symptomatic	1^st^ MRI following ARIA: resolution of ARIA-E; lecanemab resumed after 3^rd^ surveillance MRI	Yes (headaches)
30	85/F	HC	ε3/ε4	No evidence of microbleeds or vasogenic edema	Before 5^th^ infusion: 3 new ARIA-H [mild] and 2 foci ARIA-E (3.2 cm and 1.7 cm in diameter) [moderate]; lecanemab held	1^st^ MRI following ARIA: 2 new foci ARIA-H for total of 5 [moderate] and decreased size of ARIA-E [moderate]; lecanemab held; lecanemab discontinued after 4^th^ infusion	No/Yes after 4^th^ infusion
31	74/F	HTN, HC	ε4/ε4	No evidence of microbleeds or vasogenic edema	Before 5^th^ infusion: 1 new focus ARIA-H [mild] and 1 focus ARIA-E (3.4 cm in diameter) [mild]; lecanemab continued	1^st^ MRI following ARIA: 1 new focus ARIA-H for total of 2 [mild] and increased size of ARIA-E (4.1 cm in diameter) [mild]; lecanemab continued; 5^th^ surveillance MRI: 8 new ARIA-H for total of 10 [moderate] and resolution of ARIA-E; 7^th^ surveillance MRI: no change in ARIA-H [moderate]; lecanemab resumed after 7^th^ surveillance MRI	No
32	79/F	HC, DM	ε3/ε4	No evidence of microbleeds or vasogenic edema	Before 5^th^ infusion: 1 new focus ARIA-H [mild]; lecanemab continued	1^st^ MRI following ARIA: no change in ARIA-H [mild]	No
33				1 focus cerebral microbleed	Before 7^th^ infusion: 2 new foci ARIA-H for total of 3 [mild]; lecanemab continued	1^st^ MRI following ARIA: 2 new foci ARIA-H for total of 5 [moderate]; lecanemab held; 3^rd^ surveillance MRI: no change in ARIA-H [moderate]; lecanemab discontinued after 6^th^ infusion	No/Yes after 6^th^ infusion
34	69/F	HC	ε3/ε4	No evidence of microbleeds or vasogenic edema	Before 14^th^ infusion: 7 new ARIA-H [moderate] and 1 new ARIA-E (sulcal effusion) [mild]; lecanemab held		No
35	63/M	HC	ε4/ε4	No evidence of microbleeds or vasogenic edema	Before 5^th^ infusion: 1 focus ARIA-E (less than 1 cm in diameter) [mild]; lecanemab held since symptomatic	1^st^ MRI following ARIA: resolution of ARIA-E; lecanemab discontinued after 4^th^ infusion	Yes (smelling gas odor, weak, clammy, fatigue)/Yes after 4^th^ infusion
36	84/F	HTN, HC, CV	ε2/ε4	No evidence of microbleeds or vasogenic edema	Before 7^th^ infusion: 1 new ARIA-H [mild]; lecanemab continued	1^st^ MRI following ARIA: no change in ARIA-H [mild]	No
37	79/F	HTN	ε3/ε3	No evidence of microbleeds or vasogenic edema	Before 5^th^ infusion: 2 foci of ARIA-H (superficial siderosis) [moderate]; lecanemab held	1^st^ MRI following ARIA: no change in ARIA-H [moderate]; lecanamab resumed	No
38	78/F	HC	ε3/ε4	No evidence of microbleeds or vasogenic edema	Before 5^th^ infusion: 2 new foci ARIA-H [mild] and 4 new foci ARIA-E (3.0 cm, 1.8 cm, 1.5 cm, and 1.0 cm in diameter) [moderate]; lecanemab held		No/Yes after 4^th^ infusion
39			ε3/ε4	No evidence of microbleeds or vasogenic edema	Before 5^th^ infusion: 1 new focus ARIA-H (superficial siderosis) [mild]; lecanemab continued	1^st^ MRI following ARIA: resolution of ARIA-H; lecanemab discontinued after 6^th^ infusion	No/Yes after 6^th^ infusion

ARIA-H: amyloid-related imaging abnormalities-hemorrhage.

ARIA-E: amyloid-related imaging abnormalities-edema.

HTN: hypertension.

HC: hypercholesterolemia.

DM: diabetes mellitus.

CV: cardiovascular disease excluding hypertension.

**Table 4:** Baseline characteristics of patients with and without amyloid-related imaging abnormalities (ARIA).

	OVERALL	NO ARIA	ANY ARIA	P-VALUE	SOLITARY ARIA-E	P-VALUE	SOLITARY ARIA-H	P-VALUE
**N**	175	136	39		22		30	
**AGE (MEAN (SD))**	73.18 (6.19)	72.99 (6.24)	73.87 (6.02)	0.432^1^	72.50 [69.50, 76.75]	0.725^1^	74.00 [71.00, 79.00]	0.202^1^
**SEX (%)**				0.627^2^		>0.999^2^		0.975^2^
**MALE**	55 (31.4)	41 (30.1)	14 (35.9)		7 (31.8)		10 (33.3)	
**FEMALE**	120 (68.6)	95 (69.9)	25 (64.1)		15 (68.2)		20 (66.7)	
**RACE (%)**				0.254^3^		0.559^3^		0.162^3^
**CAUCASIAN**	169 (96.6)	132 (97.1)	37 (94.9)		21 (95.5)		28 (93.3)	
**AA**	4 (2.3)	2 (1.5)	2 (5.1)		1 (4.5)		2 (6.7)	
**ASIAN**	2 (1.1)	2 (1.5)	0 (0.0)		0 (0.0)		0 (0.0)	
**MMSE SCORE (MEDIAN [IQR])**	25.00 [23.00, 27.00]	25.00 [23.00, 27.00]	24.00 [23.75, 26.00]	0.155^1^	24.00 [24.00, 26.00]	0.543^1^	24.00 [24.00, 25.00]	0.187^1^
**APOE** ε**4 STATUS (%)**				**0.002**^2^		**0.041**^2^		**0.004**^2^
**HOMOZYGOTE**	25 (14.3)	13 (9.6)	12 (30.8)		7 (31.8)		10 (33.3)	
**HETEROZYGOTE**	95 (54.3)	75 (55.1)	20 (51.3)		10(45.5)		14 (46.7)	
**NON-CARRIER**	55 (31.4)	48 (35.3)	7 (17.9)		5 (22.7)		6 (20.0)	
**BASELINE MICROBLEEDS (%)**				0.082^2^		0.721^2^		0.051^2^
**NO**	155 (88.6)	124 (91.2)	31 (79.5)		19 (86.4)		23 (76.7)	
**YES**	20 (11.4)	12 (8.8)	8 (20.5)		3 (13.6)		7 (23.3)	
**BASELINE # OF MICROBLEEDS (MEDIAN [IQR])**	0.00 [0.00, 0.00]	0.00 [0.00, 0.00]	0.00 [0.00, 0.00]	**0.041**^1^	0.00 [0.00, 0.00]	0.747^1^	0.00 [0.00, 0.00]	**0.022**^1^
**BASELINE INFARCTS (%)**				0.517^3^		0.69^3^		0.711^3^
**NO**	161 (92.0)	126 (92.6)	35 (89.7)		20 (90.9)		27 (90.0)	
**YES**	14 (8.0)	10 (7.4)	4 (10.3)		2 (9.1)		3 (10.0)	
**HIGHER EDUCATION (%)**				0.318^2^		0.476^2^		0.834^2^
**NO**	40 (23.5)	28 (21.4)	12 (30.8)		7 (31.8)		8 (26.7)	
**YES**	130 (76.5)	103 (78.6)	27 (69.2)		15 (68.2)		22 (73.3)	
**HYPERTENSION (%)**				0.396^2^		0.103^2^		0.931^2^
**NO**	95 (54.3)	71 (52.2)	24 (61.5)		16 (72.7)		17 (56.7)	
**YES**	80 (45.7)	65 (47.8)	15 (38.5)		6 (27.3)		13 (43.3)	
**DYSLIPIDEMIA (%)**				0.699^2^		>0.999^2^		0.599^2^
**NO**	43 (24.6)	32 (23.5)	11 (28.2)		5 (22.7)		9 (30.0)	
**YES**	132 (75.4)	104 (76.5)	28 (71.8)		17 (77.3)		21 (70.0)	
**CARDIOVASCULAR (%)**				0.103^2^		**0.023**^2^		0.331^2^
**NO**	118 (67.4)	87 (64.0)	31 (79.5)		20 (90.9)		23 (76.7)	
**YES**	57 (32.6)	49 (36.0)	8 (20.5)		2 (9.1)		7 (23.3)	
**DIABETES (%)**				0.767^2^		0.758^2^		>0.999^2^
**NO**	143 (81.7)	110 (80.9)	33 (84.6)		19 (86.4)		25 (83.3)	
**YES**	32 (18.3)	26 (19.1)	6 (15.4)		3 (13.6)		5 (16.7)	
**DIAGNOSIS (%)**				>0.999^2^		0.79^2^		>0.999^2^
**MCI**	104 (59.4)	81 (59.6)	23 (59.0)		12 (54.5)		18 (60.0)	
**EARLY AD**	71 (40.6)	55 (40.4)	16 (41.0)		10 (45.5)		12 (40.0)	
**BMI (MEDIAN [IQR])**	25.43 [22.68, 28.55]	25.73 [23.04, 28.61]	24.72 [22.20, 27.94]	0.402^1^	23.74 [21.87, 28.06]	0.329^1^	24.24 [22.17, 27.27]	0.111^1^

1 = Kruskal-Wallis Test

2 = Chi-Squared Test

3 = Fisher’s Exact Test AA: African American MCI: mild cognitive impairment AD: Alzheimer’s disease BMI: body mass index.

Five (12.8 %) patients with ARIA were symptomatic, necessitating lecanemab suspension (Table 3). Three of these symptomatic patients were ε4 homozygous, and two were ε4 heterozygous. Patient #6 had headaches when ARIA was detected on the surveillance brain MRI before the 5^th^ infusion. The lecanemab was permanently suspended, and the headaches resolved after the discontinuation of lecanemab. Patient #7 experienced headaches after the 17^th^ infusion. Due to persistent ARIA-E, the lecanemab was permanently suspended. The headaches subsequently resolved. Patient #10 had ARIA detected before the 5^th^ infusion at which time the patient was complaining of fatigue and disorientation. Due to her ε4 homozygous status, the lecanemab was temporarily suspended and the symptoms resolved. The patient resumed lecanemab after the 4^th^ MRI following ARIA, and no further symptoms occurred. Patient #29 had headaches when the surveillance brain MRI was performed before the 7^th^ infusion. The lecanemab was held, and the headaches resolved. The lecanemab was subsequently resumed after the third surveillance MRI, with no associated side effects. Patient #35 experienced numerous symptoms of smelling a gas odor, weakness, clamminess, and fatigue when ARIA was detected on the surveillance brain MRI was performed before the 5^th^ infusion. Due to these symptoms, the lecanemab was permanently suspended.

**Fig. 2:**
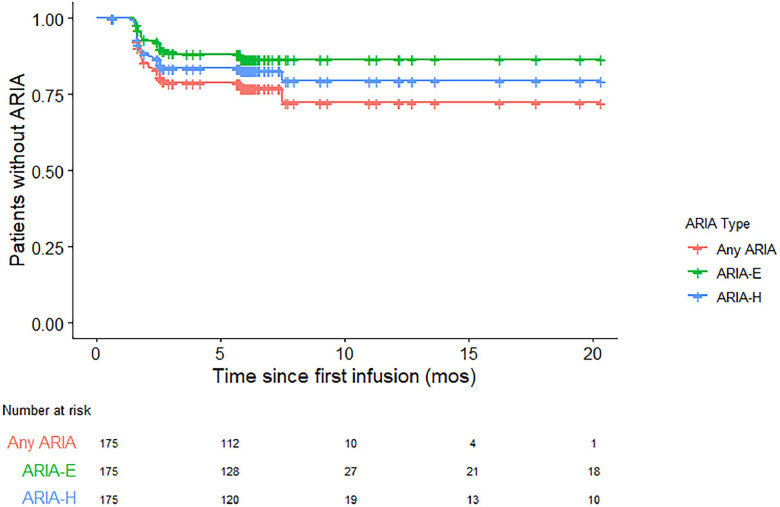
The percentage of patients without evidence of ARIA over a two-year duration.

ARIA was most commonly detected on the surveillance brain MRI performed before the 5^th^ infusion (29 [74.4 %] patients) (Fig. 2). Of the patients who underwent at least one surveillance brain MRI and had baseline MMSE testing, there were no significant differences in the rates of any ARIA (both ARIA-H and ARIA-E) (p = 0.545), solitary ARIA-H (p = 0.43), or solitary ARIA-E (>0.999) or patients who were symptomatic with ARIA (p = 0.493) comparing patients with the lowest quartile (≤ 23; n = 38 [28 %]) to those with the highest quartile (≥ 27; n = 37 [27 %]) of MMSE scores. A multivariate regression revealed that diagnosis (MCI versus mild dementia) was not a significant predictor of symptomatic ARIA (OR = 2.034 [0.311–16.424], p-value = 0.457). Additionally, MMSE score was not a significant predictor of symptomatic ARIA (OR = 1.108 [0.762-1.63], p-value = 0.588).

### 3.4 Infusion-related side effects

A total of 60 (32.1 %) patients experienced infusion-related adverse effects, 54 (90.0 %) of which occurred after the first lecanemab infusion (Table 2). Mild and transient headaches were most common, affecting 26 (48.1 %) of these patients after the first infusion. The side effects were reported either at the infusion center [0–3 h] (14 [25.9 %] patients) or between 3–24 h post-infusion (36 [66.7 %]). Of the 42 patients who received lecanemab before the pre-infusion cocktail was implemented, 19 (45.2 %) experienced an infusion-related adverse event (Table 2). Of the 145 patients who were treated with lecanemab after the pre-infusion cocktail was incorporated, 41 (28.3 %) had an infusion-related adverse event.

### 3.5 Status of lecanemab at the two-year mark of its use at our Memory Center

A total of 155 (82.9 %) patients continued to receive infusions at the two-year mark, while 32 (17.1 %) had discontinued lecanemab (Table 2). The mean number of lecanemab infusions per patient completed at the two-year mark was 24 (Range: 1-47). No patient elected to stop therapy after 18 months; all have continued infusions either every 2 or 4 weeks. Of the 32 patients who discontinued lecanemab, 10 (31.2 %) stopped lecanemab due to cognitive decline, delusions, and/or agititation associated with progression of the AD disease and 9 (28.1 %) discontinued lecanemab due to ARIA progression. Of these latter 9 patients, 4 were ε4 homozygous, 4 were ε4 heterozygous, and 1 was a ε4 non-carrier. Other reasons for suspending lecanemab are described in Table 2. Two patients died who were treated with lecanemab. One death occurred within several hours of the first infusion and was most likely cardiac-related. This case was described in detail within our initial lecanemab publication.^13^ The second patient, an 80-year-old female who was ε4 heterozygous with a history of dyslipidemia, had no evidence of ARIA on surveillance brain MRIs. After her 15^th^ and 16^th^ infusions, she was evaluated in the Emergency Department for aggression and hallucinations. These symptoms were most likely due to progressive dementia. The lecanemab was discontinued after her 19^th^ infusion. Two months later, the patient experienced a seizure and lost consciousness, was admitted to the hospital for 5 days, and was diagnosed with atrial fibrillation, a left bundle branch block, and a urinary tract infection. She died one month later.

Of the 73 patients who underwent MMSE testing prior to starting lecanemab and after the 1-year mark, 51 (69.9 %) had decreased MMSE scores, 13 (17.8 %) had increased MMSE scores, and 9 (12.3 %) had the same score (Table 2). Of the 13 patients whose MMSE scores increased from baseline to 1-year post-lecanemab, 7 had increased by one point, 3 by 2 points, 1 by 3 points, and 2 by 4 points. Of the 7 patients whose scores increased by more than one point, 4 were ε4 non-carriers, 2 were ε4 heterozygous, and 1 was ε4 homozygous. Comparing the 51 patients with a decreased MMSE score after 1-year post-infusion to the 22 patients with no decrease in MMSE scores (the MMSE score either stayed the same or improved), there was no significant association between any of the variables (patient age, sex, ApoE status, any ARIA, ARIA-H, and ARIA-E) and a decrease in MMSE scores (Supplemental Table 1). A multinomial binomial regression predicting the decrease in MMSE scorers controlling for age, gender, and ApoE status revealed that none of the variables were significant predictors for a decrease in MMSE scores (Supplemental Table 2) or a change in MMSE scores (Supplemental Table 3). Supplemental Figure 1 depicts a spaghetti plot for all 73 patients who had baseline MMSE testing and then after 1-year of lecanemab infusions. Supplemental Figure 2 highlights a spaghetti plot for the patients who developed ARIA and who had baseline MMSE testing and after 1-year of infusions.

Of the 21 patients who underwent more than 40 infusions (a minimum of 18 months), 16 had MMSE scores performed at baseline and after 1-year post-lecanemab (Supplemental Figure 3). Of these 16 patients, 12 had decreased MMSE scores, 3 had increased scores, and the score remained in the same in 1 patient. One patient’s MMSE score increased by 4 from a baseline score of 20 to a subsequent score of 24 after 16 months of lecanemab infusions. The patient was 59 years old when he initiated lecanemab, had undergone 41 infusions, was a ε4 non-carrier, and had no evidence of ARIA or other adverse side effects of the drug.

## 4 Discussion

### 4.1 Data presented

Recent studies have reported the benefits of lecanemab while warning of the potential risks of ARIA, especially in patients who are ε4 homozygous [17,18]. Paczynski and colleagues reported 234 patients treated with lecanemab in a specialty memory clinic over 14 months [18]. Of the 194 patients who underwent at least one surveillance brain MRI, 42 (22 %) developed ARIA, 11 (26.2 %) of whom were symptomatic. In Bregman and colleagues’ study of 86 patients treated with lecanemab in a Cognitive Neurology Unit in a tertiary hospital setting, 16 (18.6 %) patients developed ARIA, most of whom were asymptomatic [17]. Bregman et al. excluded patients homozygous for APOE ε4 and, therefore, rates of ARIA would be expected to be lower overall. Our study concurs with these studies, reflected by 20 % of patients who had ARIA detected on surveillance brain MRIs, half of whom were ε4 homozygous and 30 % of whom were ε4 heterozygous. Five patients were symptomatic when ARIA was identified, resulting in a suspension of lecanemab. Three of these symptomatic patients were ε4 homozygous, and two were ε4 heterozygous. ARIA was most commonly detected on the surveillance brain MRI performed before the 5^th^ infusion (29 [74.4 %] patients). These findings confirm the relatively high risk of ARIA in patients treated with lecanemab, especially in those who are ε4 homozygous or heterozygous. These particular APOE ε4 statuses also portend a higher likelihood of symptomatic ARIA and discontinuing lecanemab due to ARIA progression. It is important to discuss the risks and benefits of starting lecanemab with patients and their families, especially in those who are ε4 homozygous. Unlike Paczynski and colleagues’ study, our study did not demonstrate a relationship between the MCI/mild dementia diagnosis and the lowest quartile of baseline MMSE scores with the development of ARIA. However, both of our studies reported that isolated ARIA-H was more frequent in patients with higher numbers of microbleeds on baseline brain MRI. In our study, patients with more baseline microbleeds more frequently developed any ARIA (ARIA-H and ARIA-E) and solitary ARIA-H. The presence of baseline microbleeds was also associated with a higher frequency of solitary ARIA-H. Of the 73 patients who underwent MMSE testing prior to starting lecanemab and after the 1-year mark in our study, 51 (69.9 %) had decreased MMSE scores, 13 (17.8 %) had increased MMSE scores, and 9 (12.3 %) had the same score.

In Paczynski et al.’s study, 87 (37 %) patients experienced infusion-related reactions, with 92 % occurring following the first or second infusion [18]. These reactions were rated as mild (67 %) or moderate (28 %). Their treatment protocol was changed after observing the high number of infusion-related reactions. Patients were subsequently pre-treated with loratadine and acetaminophen before the initial infusion. In Bregman and colleagues’ study, 19 (22.1 %) patients experienced mild and transient infusion-related reactions [17]. The number, severity, and timing of infusion-related side effects in our study is comparable to these studies. Sixty (32.1 %) patients experienced infusion-related adverse effects, 54 (90.0 %) of which occurred after the first lecanemab infusion and half of which were mild and transient headaches. Over 90 % of the side effects were reported at the infusion center or within 24 h post-infusion.

In Paczynski et al.’s study, 23 (9.8 %) patients discontinued lecanemab, 10 [43.5 %] for ARIA [18]. Bregman et al. reported that 17 (19.8 %) patients withdrew from treatment, with 5 [29.4 %] attributing ARIA as the reason [17]. A total of 32 (17.1 %) patients had discontinued lecanemab at the 2-year mark in our study, with 9 [28.1 %] ending their treatment due to ARIA progression.

### 4.2 Continued challenges with lecanemab at our Memory Center

We previously described the numerous insurance coverage challenges [13], and these hurdles have persisted (Table 5). The main obstacles are the initial approval and re-authorization of lecanemab by insurance companies, with different requirements for various Medicare options and commercial insurance. Original Medicare approves lecanemab for one year, while Medicare Advantage and commercial insurance plans approve this drug for 6 months. The authorization team at our Memory Center automatically submits the lecanemab re-approval request to insurance companies. Several patients have missed their scheduled infusions since the re-authorizations have not been approved in a timely manner. Some insurance companies require documentation that a patient is tolerating lecanemab well and is benefiting from this medication. These companies also necessitate a statement that the patient has had no evidence of moderate or severe ARIA on surveillance brain MRIs and that the patient has no clinical presentations suggesting ARIA.

**Table 5:** Key insights gained from our two-year experience with lecanemab.

Challenges with lecanemab	Key insights
Initial approval and re-authorization of lecanemab by insurance companies	Importance of shared decision-making progress between patient, caregiver, and provider to initiate lecanemab infusion
Brain MRI scheduling concerns due to many patients who require brain imaging	Providers offer anti-amyloid therapy early in the evaluation process
	Some patients do not want to transition to lecanemab maintenance every 4 weeks instead of every 2 weeks after 18 months of lecanemab infusions
	Pre-infusion oral cocktail (acetaminophen 650 mg, loratadine 10 mg, and famotidine 20 mg) decreased infusion-related adverse events
	Reduced post-infusion observation time is possible without safety concerns

We also continue to experience issues with MRI scheduling due to many patients who require brain imaging and challenges with MRI scan capacity. To overcome this obstacle, we instituted a new MRI software program Deep Resolve (Siemens Healthineers; Malvern, PA) in January 2025 that increased the speed of each brain MRI scan by 70 % [19]. Adopting this new program has lessened the MRI workload and has permitted a greater number of scans to be performed.

### 4.3 Key insights gained from our two-year experience with lecanemab

Through our 2-year experience with lecanemab, we emphasize the importance of a shared decision making process between the patient, caregiver, and provider to initiate lecanemab infusion (Table 5). Patients and their caregivers are thoughtful and well-informed in the decision-making process, as they weigh the risks of lecanemab versus the frequency of infusions. Our Memory Center’s comfort level with treating patients with lecanemab has increased over the 2-year duration. This enhanced confidence has allowed us to lighten the patient restrictions as set forth in the lecanemab Appropriate Use Recommendations [2]. Patients who were previously excluded from using lecanemab due to a lifetime history of seizures and using other monoclonal antibodies such as denosumab are now potentially able to receive lecanemab through a shared decision-making process between the individual treating clinician and patient.

This theme of individualized decisions has been reported in association with lecanemab [20,21]. Parks and colleagues performed a qualitative study of 22 patients with AD highlighting how patients balance the risks and benefits of lecanemab [21]. Numerous considerations were contemplated prior to initiating lecanemab, including individual characteristics, family factors, trust in the provider, insurance coverage, and corresponding with patients who are receiving lecanemab. In Parks et al.’s qualitative study of 27 clinicians who prescribe lecanemab across seven medical centers, the techniques of how clinicians describe the risks and benefits of lecanemab vary, with most focusing on patient comorbidities, eligibility criteria fit, and degree of social support or family involvement in making decisions [20]. Few clinicians use patients’ goals to guide the conversation, and some may not recommend lecanemab and even may advise against it [20].

Our Memory Center providers try to offer anti-amyloid therapy as a possible treatment option early in the evaluation process among appropriate candidates so that the patients and their caregivers have adequate time to make an informed decision. The patient’s first appointment consists of a detailed history, physical exam, cognitive testing (typically MMSE or MOCA), blood work (CBC, CMP, vitamin B12, and TSH), screening for additional nutritional deficiencies, and a brain MRI. Patients undergo formal neuropsychology testing on a case by case basis. CSF studies, blood-based biomarkers, or amyloid PET studies are then performed to confirm AD pathology. Patients subsequently undergo APOE genotyping and continue to discuss the possible benefits and risks of anti-amyloid medications to help them make an informed decision.

At 18 months of lecanemab infusions, patients have the option of transitioning to lecanemab maintenance every 4 weeks. In our Memory Center, five patients started the monthly lecanemab maintenance after 18 months of biweekly infusions and then requested to revert to every 2 week infusions. These patients and their families attributed subjective cognitive decline for their reason to resume the infusions every 2 weeks. Two additional patients did not want to switch to the monthly maintenance and continued on the every 2-week regimen. While we are unable to explain why patients experience subjective cognitive decline with lecanemab administered every 4 weeks compared to every 2 weeks, we posit that patients may thrive on the socialization by coming to the infusion center where they receive encouragement in a positive environment. In our experience, no insurance companies have denied covering the biweekly infusions after 18 months.

In our initial lecanemab publication, 26 (37 %) patients experienced infusion-related side effects after their first lecanemab infusion, primarily headaches and shaking/chills/rigors [13]. Twenty-three (88 %) of these 26 patients reported the side effects either at the infusion center [0–3 h] (10 patients) or between 3–24 h post-infusion (13 patients). The side effects following the first lecanemab infusion mainly occurred in the first 3 months of prescribing lecanemab at our Memory Center. Similar to Paczynski and colleagues, we implemented a pre-infusion cocktail on December 31, 2023 which subsequently plummeted the number of post-infusion side effects. The primary difference between our protocol and theirs is that they pre-treat patients only for the initial infusion, while we pre-treat patients prior to every lecanemab infusion. As we continued to observe a decline in side effects following lecanemab, we revised the duration of post-infusion observation in August 2025. We reduced the observation period after the 1^st^ infusion from 3 to 2 h, after the 2^nd^ infusion from 2 h to 1 h, and all subsequent monitoring periods to 30 min.

### 4.4 Strengths and limitations of the current study

The strength of the present study is the large number of patients with MCI or mild dementia who were treated with lecanemab in a regional community medical center over a 2-year duration. We have incorporated the lessons learned from our initial experience with lecanemab in the first 6 months and expanded upon our knowledge in the subsequent 18 months. We continue to face similar challenges with insurance and brain MRI scheduling, however, we have implemented measures to mitigate these obstacles. With the imminent implementation of subcutaneous lecanemab infusions at our Memory Center, the care of patients with MCI and mild dementia will be transformed.

The limitations of the current study are its retrospective nature and variability of the reports among the radiologists who reviewed the baseline and surveillance brain MRIs. Our Memory Center has recently employed the use of machine learning capabilities that assist the radiologists in detecting ARIA on the baseline and surveillance brain MRIs which will expedite and standardize methods of quantitatively determining the presence and extent of ARIA. Another limitation is that not all patients underwent MMSE testing prior to starting lecanemab to permit a comparison between scores at baseline and after treatment. It is interesting to note that 30 % of patients who underwent MMSE testing prior to starting lecanemab and between the 1 and 2 year mark had either increased MMSE scores or had the same score.

In conclusion, the main aim of the present study was to define our experience with lecanemab with key insights and to document the incidence of ARIA detected on surveillance brain MRIs over a two-year period. We also wanted to determine how many patients discontinued lecanemab and their particular reasons. Our findings suggest that ARIA is a significant concern especially in patients who are ε4 homozygous. Comprehensive discussions among the patient, caregiver, and provider are valuable in deciding whether lecanemab is an appropriate and beneficial drug.

## Funding

The authors declare that there was no funding for their study.

## Declaration of generative AI and AI-assisted technologies in the writing process

AI has not been used at all in the preparation of this manuscript.

## Data statement

All data supporting the findings of this study are available within the paper.

## CRediT authorship contribution statement

**Lisa B.E. Shields:** Writing – review & editing, Writing – original draft, Visualization, Validation, Supervision, Software, Resources, Project administration, Methodology, Investigation, Formal analysis, Data curation, Conceptualization. **Hust Hannah:** Writing – review & editing, Software, Resources, Methodology, Investigation, Formal analysis, Data curation, Conceptualization. **Gregory E. Cooper:** Writing – review & editing, Supervision, Project administration, Investigation, Formal analysis, Data curation, Conceptualization. **Theresa Kluthe:** Writing – review & editing, Visualization, Validation, Software, Resources, Methodology, Investigation, Formal analysis, Data curation. **Rachel N. Hart:** Writing – review & editing, Investigation, Formal analysis, Data curation, Conceptualization. **Andrew P. Thaliath:** Writing – review & editing, Investigation, Formal analysis, Data curation, Conceptualization. **Brandon C. Dennis:** Writing – review & editing, Investigation, Formal analysis, Data curation, Conceptualization. **Stephanie W. Freeman:** Writing – review & editing, Investigation, Formal analysis, Data curation, Conceptualization. **Jessica F. Cain:** Writing – review & editing, Investigation, Formal analysis, Data curation, Conceptualization. **Whoy Y. Shang:** Writing – review & editing, Investigation, Formal analysis, Data curation, Conceptualization. **Kendall M. Wasz:** Writing – review & editing, Investigation, Formal analysis, Data curation, Conceptualization. **Adam T. Orr:** Writing – review & editing, Visualization, Validation, Software, Resources, Investigation, Formal analysis, Data curation, Conceptualization. **Christopher B. Shields:** Writing – review & editing, Visualization, Validation, Supervision, Software, Resources, Project administration, Methodology, Investigation, Formal analysis, Data curation, Conceptualization. **Shirish S. Barve:** Writing – review & editing, Investigation, Formal analysis, Data curation, Conceptualization. **Kenneth G. Pugh:** Writing – review & editing, Visualization, Validation, Supervision, Software, Resources, Project administration, Methodology, Investigation, Formal analysis, Data curation, Conceptualization.

## Declaration of competing interest

The authors declare the following financial interests/personal relationships which may be considered as potential competing interests: Gregory E. Cooper, MD, PhD receives research funding from Eisai, Lilly, Novartis, and Davos Alzheimer’s Collaborative. Rachel N. Hart, DO is on the Speaker’s Bureau of Lilly. The rest of the authors declare that they have no known competing financial interests or personal relationships that could have appeared to influence the work reported in this paper.

## Appendix Supplementary materials

Image, application 1

## References

[1] Alzheimer's Association (2025). 2025 Alzheimer's disease facts and figures. https://www.alz.org.

[2] Cummings J., Apostolova L., Rabinovici G.D., Atri A., Aisen P., Greenberg S. (2023). Lecanemab: appropriate use recommendations. J Prev Alzheimers Dis.

[3] Stewart J. (2025). Leqembi FDA approval history. https://www.drugs.com/history/leqembi.html.

[4] van Dyck C.H., Swanson C.J., Aisen P., Bateman R.J., Chen C., Gee M. (2023). Lecanemab in early Alzheimer's disease. N Engl J Med.

[5] Ashmawy R.E., Okesanya O.J., Ukoaka B.M., Daniel F.M., Ezedigwe S.G., Agboola A.O. (2025). Exploring the efficacy and safety of lecanemab in the management of early Alzheimer's disease: a systematic review of clinical evidence. J Alzheimers Dis.

[6] Honig L.S., Sabbagh M.N., van Dyck C.H., Sperling R.A., Hersch S., Matta A. (2024). Updated safety results from phase 3 lecanemab study in early Alzheimer's disease. Alzheimers Res Ther.

[7] Hossain M.F., Husna A.U., Kharel M. (2024). Use of lecanemab for the treatment of Alzheimer's disease: a systematic review. Brain Behav.

[8] McDade E., Cummings J.L., Dhadda S., Swanson C.J., Reyderman L., Kanekiyo M. (2022). Lecanemab in patients with early Alzheimer's disease: detailed results on biomarker, cognitive, and clinical effects from the randomized and open-label extension of the phase 2 proof-of-concept study. Alzheimers Res Ther.

[9] Qiao Y., Chi Y., Zhang Q., Ma Y. (2023). Safety and efficacy of lecanemab for Alzheimer's disease: a systematic review and meat-analysis of randomized clinical trials. Front Aging Neurosci.

[10] Swanson C.J., Zhang Y., Dhadda S., Wang J., Kaplow J., Lai R.Y.K. (2021). A randomized, double-blind, phase 2b proof-of-concept clinical trial in early Alzheimer's disease with lecanemab, an anti-Abeta protofibril antibody. Alzheimers Res Ther.

[11] Yan L., Zhang L., Xu Z., Luo Z. (2025). A real-world disproportionality analysis of FDA adverse event reporting system (FAERS) events for lecanemab. Front Pharmacol.

[12] Honig L.S., Barakos J., Dhadda S., Kanekiyo M., Reyderman L., Irizarry M. (2022). ARIA in patients treated with lecanemab (BAN2401) in a phase 2 study in early Alzheimer's disease. Alzheimer's Dement.

[13] Shields L.B.E., Hust H., Cooley S.D., Cooper G.E., Hart R.N., Dennis B.C. (2024). Initial experience with lecanemab and lessons learned in 71 patients in a regional medical center. J Prev Alzheimers Dis.

[14] Eisai (2025). FDA approves LEQMBI (lecanemab-irmb) IV maintenance dosing for the treatment of early Alzheimer's disease. https://media-us.eisai.com/2025-01-26-FDA-Approves-LEQEMBI-R-lecanemab-irmb-IV-maintenance-dosing-for-the-treatment-of-early-Alzheimers-disease.

[15] Agarwal A., Gupta V., Brahmbhatt P., Desai A., Vibhute P., Joseph-Mathurin N. (2023). Amyloid-related imaging abnormalities in Alzheimer disease treated with anti-amyloid-beta therapy. Radiographics.

[16] Cogswell P.M., Barakos J.A., Barkhof F., Benzinger T.S., Jack C.R., Poussaint T.Y. (2022). Amyloid-related imaging abnormalities with emerging Alzheimer disease therapeutics: detection and reporting recommendations for clinical practice. AJNR Am J Neuroradiol.

[17] Bregman N., Nathan T., Shir D., Omer N., Levy M.H., David A.B. (2025). Lecanemab in clinical practice: real-world outcomes in early Alzheimer's disease. Alzheimers Res Ther.

[18] Paczynski M., Hofmann A., Posey Z., Gregersen M., Rudman M., Ellington D. (2025). Lecanemab treatment in a specialty memory clinic. JAMA Neurol.

[19] Siemens Healthineers (2025). Deep Resolve. https://wws.siemens-heallthineers.com/en-us-magnetic-resonance-imaging/technologies-and-innovations/deep-resolve.

[20] Parks A.L., Thacker A., Dohan D., Gomez L.A.R., Gale S.A., Johnson K.G. (2025). Characterizing clinician communication with patients about lecanemab: a qualitative study of clinicians across seven academic medical centers. Alzheimers Dement.

[21] Parks A.L., Thacker A., Dohan D., Gomez L.A.R., Ritchie C.S., Paladino J. (2025). A qualitative study of people with Alzheimer's disease in a memory clinic considering lecanemab treatment. J Alzheimers Dis.

