# Supplementary material for: Lecanemab over a two-year duration: Key insights from a regional specialty medical center
Source: J Prev Alzheimers Dis. 2026 Jan 24;13(3):100489. doi: 10.1016/j.tjpad.2026.100489 (PMC12860706; doi:10.1016/j.tjpad.2026.100489)
Supplement: Supplementary file 1 [file mmc1.docx]

**Supplementary Table 1: MMSE Scores at Baseline and after 1-year of Lecanemab Infusions (n=73)**

| Variables | No decrease | Decrease | P-value |
| --- | --- | --- | --- |
| n | 22 | 51 |  |
| Age (mean (SD)) | 70.41 (7.31) | 72.33 (6.36) | 0.261^1^ |
| Gender (%) |  |  | 0.776^2^ |
| Male | 11 (50.0) | 22 (43.1) |  |
| Female | 11 (50.0) | 29 (56.9) |  |
| ApoE (%) |  |  | 0.814^2^ |
| Homozygous | 3 (13.6) | 5 (9.8) |  |
| Heterozygous | 12 (54.5) | 26 (51.0) |  |
| Non-Carrier | 7 (31.8) | 20 (39.2) |  |
| Any ARIA (%) |  |  | 0.289^2^ |
| No | 17 (77.3) | 45 (88.2) |  |
| Yes | 5 (22.7) | 6 (11.8) |  |
| ARIA-H (%) |  |  | 0.691^2^ |
| No | 19 (86.4) | 46 (90.2) |  |
| Yes | 3 (13.6) | 5 (9.8) |  |
| ARIA-E (%) |  |  | 0.357^2^ |
| No | 19 (86.4) | 48 (94.1) |  |
| Yes | 3 (13.6) | 3 (5.9) |  |

^1^ = ANOVA; ^2^ = Fisher’s Exact test

**Supplementary Table 2: Multinomial binomial regression predicting the decrease in MMSE, controlling for age, gender, and ApoE status**

|  | OR | 2.50% | 97.50% | P-value |
| --- | --- | --- | --- | --- |
| Age | 1.053 | 0.97 | 1.147 | 0.22 |
| Gender (Female) | 1.421 | 0.478 | 4.311 | 0.527 |
| ApoE (Heterozygous) | 0.827 | 0.133 | 4.377 | 0.826 |
| ApoE (Non-Carrier) | 1.243 | 0.189 | 7.1 | 0.809 |
| ARIA (ARIA-H) | 3.758 | 0.186 | 133.896 | 0.402 |
| ARIA (Both ARIA) | 2.721 | 0.095 | 147.6 | 0.571 |
| ARIA (No ARIA) | 5.852 | 0.495 | 141.138 | 0.175 |

**Supplementary Table 3: Multinomial linear regression predicting a change in MMSE scores, controlling for age, gender, and ApoE status**

|  | Beta | 2.50% | 97.50% | P-value |
| --- | --- | --- | --- | --- |
| Age | 0.026 | -0.081 | 0.133 | 0.629 |
| Gender (Female) | 0.014 | -1.431 | 1.459 | 0.984 |
| ApoE (Heterozygous) | -1.117 | -3.516 | 1.282 | 0.356 |
| ApoE (Non-Carrier) | -0.468 | -2.923 | 1.986 | 0.704 |
| ARIA (ARIA-H) | 2.821 | -1.564 | 7.207 | 0.203 |
| ARIA (Both ARIA) | 3.514 | -1.363 | 8.391 | 0.155 |
| ARIA (No ARIA) | 2.364 | -1.177 |  | 0.187 |

**Supplementary Figure 1: Changes in MMSE scores from baseline to after 1-year of lecanemab infusions
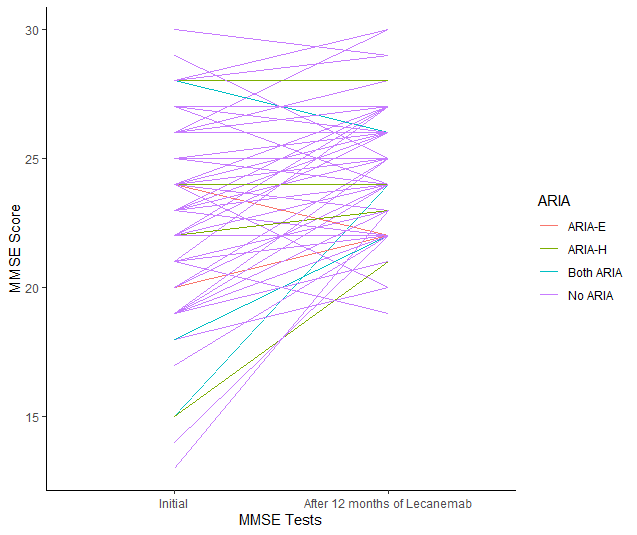
**

**Supplementary Figure 2: Changes in MMSE scores from baseline to after 1-year of lecanemab infusions in patients with ARIA detected on surveillance brain MRI scans**


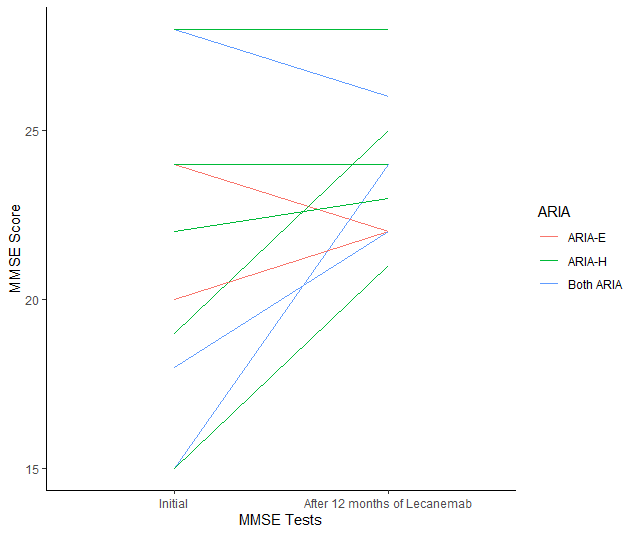


**Supplementary Figure 3:** **Change in MMSE scores from Baseline to after 40 Lecanemab Infusions**
